# Brazilian Immigrant Parents’ Preferences for Content and Intervention Modalities for the Design of a Family-Based Intervention to Promote Their Preschool-Age Children’s Healthful Energy Balance-Related Behaviors

**DOI:** 10.3390/ijerph20064817

**Published:** 2023-03-09

**Authors:** Thaís Vilasboas, Qun Le, Mary L. Greaney, Ana Cristina Lindsay

**Affiliations:** 1 Department of Biology, College of Science and Mathematics, University of Massachusetts Boston, Boston, MA 02125, USA; 2 Department of Public Health, Zuckerberg College of Health Sciences, University of Massachusetts—Lowell, Lowell, MA 01854, USA; 3 Department of Health Studies, College of Health Sciences, University of Rhode Island, Kingston, RI 02881, USA; 4 Department of Exercise and Health Sciences, Robert and Donna Manning College of Nursing and Health Sciences, University of Massachusetts Boston, Boston, MA 02125, USA

**Keywords:** energy balance-related behaviors, parents, Brazilian, immigrant, preschool, obesity, intervention

## Abstract

Brazilians are a rapidly growing ethnic immigrant population in the United States (U.S.), and there is a lack of childhood obesity prevention interventions addressing the needs of Brazilian preschool-age children. Using the family ecological model (FEM) as a guide, this developmental cross-sectional study assessed the preferences (content, intervention modality, and language) of 52 individual Brazilian immigrant parents (27 mothers, 25 fathers) for a family-based intervention to promote healthful energy balance-related behaviors (EBRB). Overall, 85% or more of parents reported being interested or very interested in content related to five of the seven assessed EBRBs (increasing fruits and vegetables, reducing unhealthy foods and sugar-sweetened beverages, increasing physical activity, and reducing screen time). Parent-preferred intervention modalities were group sessions delivered by community health workers (CHWs, 86.5%), email (84.6%), and messaging (78.8%), with most parents (71.2%) indicating a preference for content in Portuguese. Interventions integrating multiple components, such as group sessions offered by CHWs and text messaging using SMS and WhatsApp, should be considered. Future steps for intervention development should include investigating different communication channels and their integration into a culturally and linguistically tailored family-based intervention designed to promote healthful EBRBs of preschool-age children in Brazilian families living in the U.S.

## 1. Introduction

Childhood obesity is a complex public health problem disproportionally affecting ethnic minority children in immigrant families [1,2]. Promoting healthful energy balance-related behaviors (EBRBs) in young children, a fundamental obesity preventative strategy, is critical for addressing health disparities in childhood obesity among ethnic minority and immigrant populations [3,4,5]. Accumulating evidence indicates that unhealthful EBRBs, including unhealthy eating behaviors, such as high consumption of unhealthy foods and beverages high in sugar, also known as sugar-sweetened beverages [SSB], inadequate physical activity (PA), excessive sedentary behavior/screen time, and short sleep duration increase the risk of child obesity [4,5,6,7].

Early childhood is a critical period for developing health habits and presents a unique opportunity for early interventions to support children in developing healthful EBRBs [8,9]. Evidence suggests parents’ critical and unique role in promoting and maintaining health and preventing diseases [10,11]. Parents play a central role in the family as primary caregivers, primarily responsible for their children’s nutrition and PA patterns, particularly in early childhood [10,12,13,14]. Consequently, parents should be considered important forces for change in their children’s behaviors [9,15].

Parents are influential in their children developing and maintaining life-long healthful behaviors through their modeling, parenting practices, and routines they establish in the home environment [9]. Therefore, supporting parents in developing the skills to engage in healthful parenting practices and creating a family environment that promotes healthful EBRBs is critical to preventing childhood obesity [5,8,16]. Research indicates that parents, including racial/ethnic minority parents, want to learn how to support their young children in developing healthful EBRBs [8,17,18]. Hence, identifying parents’ preferences (content and modalities) for interventions to promote healthful EBRBs is essential for designing effective family-based interventions that address parents’ specific needs.

Most available family-based interventions designed to promote healthful EBRBs and prevent childhood obesity have focused on mothers [19,20]. Although mothers are often the primary parent providing care for their children, fathers also play an influential role in parenting practices and household routines that influence young children’s EBRBs [8,20]. Previous research suggests that Latino fathers in the United States (U.S.), including immigrant fathers, believe it is essential for their children to develop healthful behaviors during childhood [21,22]. Therefore, they try to model and help their children develop healthful behaviors [22,23,24,25,26]. However, research also suggests that Latino fathers may contribute to the children participating in unhealthful EBRBs, such as screen time and consuming excessive unhealthy snacks [21,22,27,28]. For example, recent research found that Latina mothers perceive fathers as negatively influencing children’s EBRBs by bringing high-calorie foods, such as pizza and SSB, home and using sweets and savory foods to reward children for positive behaviors [21,22].

Brazilians are a rapidly growing ethnic minority immigrant population in the U.S., now home to the largest population of Brazilians outside of Brazil [29]. According to the American Community Survey (ACS) data, which provides the best estimate of demographic, economic, and social characteristics of Brazilians in the U.S., about 710,000 Brazilians, including those born in Brazil and their U.S.-born descendants, resided in the United States in 2019 [29]. Nonetheless, this count may underestimate the number of Brazilians in the U.S., due to the large number of Brazilians who are undocumented [29]. Massachusetts is the state with the second largest Brazilian population in the U.S., behind only Florida. Brazilians are categorized as Latinos in national datasets (e.g., U.S. Census); hence, data specific to this population in the U.S. is limited [29].

Although Brazilians share many cultural characteristics with other Latino populations (e.g., personalism, familism, machismo, Catholicism official religion, etc.), Brazilians have distinctive characteristics, including speaking Portuguese, a very important cultural difference between Brazilians and Spanish-speaking Hispanic groups [30,31]. As a result, many Brazilians living in the U.S. do not view themselves as Hispanic, as they speak a different language (Portuguese). They also have different cultural origins (Portuguese, African, and Indigenous) that make them distinct from other Hispanic groups [30,31]. Hence, there is an urgent need to develop interventions to promote EBRBs that meet the specific needs of this unique and growing immigrant population in the U.S. It also is important to assess if mothers and fathers have the same preferences for interventions. This will ensure the design of salient interventions for both Brazilian immigrant mothers and fathers. To date, no interventions promoting healthful EBRBs to prevent childhood obesity have addressed the specific needs of Brazilian immigrants in the U.S. Therefore, the present descriptive study was conducted as formative research to assess Brazilian immigrant parents’ preferences for informational content, delivery modality, and access and use of communication technology for the development of a family-based intervention to promote healthful EBRBs and prevent child obesity [32,33,34,35,36].

## 2. Materials and Methods

### 2.1. Theoretical Model

Using the family ecological model (FEM) [37], this developmental [32,33,34,35,36] cross-sectional pilot study [31] was conducted to inform the design of a family-based early childhood obesity prevention intervention. The FEM builds on the ecological system theory (EST), which posits that human behavior cannot be understood without considering the contexts in which it occurs [38]. The EST emphasizes the individual as a focal intervention point while considering the broader social context of influences [38]. The FEM was developed to emphasize the importance of focusing on the family—rather than the individual—as the focal point of the intervention target. The inner circle summarizes how parents influence children’s diet, activity, and screen-based behaviors [37]. These processes include parents’ knowledge and beliefs about obesity, the modeling of healthy behaviors., and the opportunities they create for healthy eating and physical activity. Research documents the important role of these factors in predicting children’s lifestyle behaviors [37]. The outer domains of the FEM represent theoretically justifiable contextual factors, including demographic factors and child characteristics.

### 2.2. Participants

Eligibility criteria to participate in this study included: (a) self-identify as Brazilian; (b) with at least one child between the ages of 2 and 5 years; (c) ≥21 years of age; (d) living in Massachusetts for at least 12 months; (e) speaking Portuguese or English; and (f) willing to provide informed consent [31].

### 2.3. Data Collection

Data were collected between January and August 2020 in selected Massachusetts communities with sizeable Brazilian immigrant populations [31,39]. We used both direct (e.g., in-person outreach at faith- and community-based events, such as meeting participants after church services, at faith-based community events, and outreach to personal contacts of research staff and recruiting partners) and indirect (e.g., posting flyers at local Brazilian businesses and community-based social and health services agencies, attending events and making announcements at predominantly Brazilian churches, and also using social media, such as Facebook postings) recruitment strategies to enroll study participants [31,39]. All in-person recruitment was conducted by bilingual, bicultural, and trained research assistants who were native Brazilians, undergraduate students in health-related disciplines [39]. In addition, we recruited participants through a “word of mouth” or snowball sampling approach by asking participants enrolled in the study to ask their Brazilian friends if they would be interested in participating [31,39]. Interested individuals called the phone number included on the flyer or spoke with study staff at church events [31,39].

Three authors developed a survey instrument in English based on a review of the literature and the “5 − 2 − 1 − 0 + 10” public health recommended lifestyle and obesity prevention goals, as well as our previous qualitative research with multi-ethnic Latinos [22,40,41,42,43] and Brazilian parents living in the U.S. by the research team [4,6,44,45,46,47,48,49]. The core survey included 44 items divided into three sections [22,31,40]. The original survey instrument developed in English was translated (Spanish and Portuguese) and pilot-tested for use with Hispanics [50,51] and Brazilians [31]. The first section had six items assessing parents’ perception of the importance of EBRBs for their preschool-aged children framed in terms of current public health recommendations (3 on healthy eating behaviors [promoting consumption of five or more fruits and vegetables per day, reducing consumption of unhealthy/junk foods, and reducing consumption of SSB, and 3 items on healthy 24 h movement behaviors [promoting at least 90 min of daily PA, limiting screen time to less than two h/day, and promoting 10 h sleep/night]) [31,50,51]. The second section included items assessing sources used by parents to obtain information to support EBRBs of their preschool-aged children (12 items). The third section included 26 items assessing parents’ preferences for a family-based intervention to promote healthful EBRBs, including content (7 items), delivery channels (11 items), access and use of communication technology (7 items), and language preference (1 item) [31,50,51].

Additionally, the survey included a section with two items assessing parents’ self-identified weight and their perceptions of their preschool-age child’s weight status (underweight, normal weight, overweight, obese), a section on sociodemographic characteristics (age, marital status, country of birth, years of residency in the U.S., primary language spoken, educational attainment, and annual household income) used in several of our previous studies with Brazilian immigrants in the U.S. [31,46,47,48,49,50,51], access and type of health insurance, and participation in the Special Supplemental Nutrition Program for Women, Infants, and Children (WIC) [31,46,47,48,49,50,51]. Finally, the survey included the Short Acculturation Scale for Hispanics (SASH), a 12-item scale assessing participants’ acculturation level [52] that was used in our previous studies with Brazilian parents [31,46,47,48,49]. As recommended by the SASH developers, acculturation scores were computed by averaging across the 12 items, measured on a scale of 1 to 5, and scores were then dichotomized (high vs. low) [31,52,53]. We used the recommended cutoff point scores to categorize respondents as having a low acculturation level (SASH < 2.99) or a high acculturation level (SASH ≥ 2.99) [31,52].

The final survey was translated into Portuguese by two trained public health professionals and native Portuguese (Brazilian) speakers [31]. The survey was pilot tested with three Brazilian parents prior to use in the current study, and these results are not included in this study [31]. Parents with more than one preschool-age child were asked to think of the oldest age-eligible child when answering the survey questions [31]. The average time for completing the survey was 15 min [31]. Participants received a USD 25 gift card at the end of the interview for their participation [31].

### 2.4. The Current Study

This paper presents data derived from participants’ responses to questions from the third section of the survey (see above) [31]. Analysis of data from Section 1 and Section 2 have been published elsewhere [31]. In addition, data derived from responses about participants’ socio-demographics, access and type of health care insurance, acculturation, and parents reported perceived self and child’s weight status were used in the analysis [31].

Preferences for informational content were assessed by the following seven questions: “The next questions are about how much you would be interested in learning more about the following topics related to your child’s preschool-age health. Help your child: (1) eat more healthy foods such as fruits and vegetables, etc., (2) eat less unhealthy or “junk” (e.g., chocolates, candy, etc.) and fast food (e.g., hamburgers, fries, etc.), (3) drink less sugar-sweetened beverages (e.g., soft drinks, artificial juices, etc.), (4) drink more water, (5) be more physically active, (6) reduce the use of electronics (e.g., television, iPad, iPhone, computer, video game, etc.) or have less screen-time, (7) have adequate sleep every night (i.e., >10 h/night)” (5-point Likert scale: 1 = not interested to 5 = very interested) [31,50,51].

Preference for delivery channels or intervention modalities (11 items) was measured by the following questions: “If you were to enroll in a health promotion program for parents of preschool-age children, what would be your preference to receive such information?” (5-point Likert scale: 1 = completely disagree—5 = completely agree): (1) email, (2) text or SMS, (3) WhatsApp, (4) social media (e.g., Facebook, Instagram, Pinterest, etc.), (5) English language website, (6) Portuguese language website, (7) telephone calls by trained community health workers (CHWs), (8) individual sessions delivered by trained CHWs, (9) short duration courses offered by trained CHWs, (10) short-duration courses offered by trained parents like me or peer parents, and 11) printed materials [31,50,51]. Access and use of technology (7 items) was measured by the following questions: “Do you have a computer at home?” (yes, no); “Do you have access to Internet at home?” (yes, no); “How often do you check your email?” (daily, more than once a week, less than once a week, once or less a month); “Do you have a mobile telephone where you can receive SMS/Text?” (yes, no); “Do you use WhatsApp?” (yes, no); “Do you use social media (e.g., Facebook, Instagram, Pinterest)” (yes, no); “How often do you check messages on your WhatsApp, SMS/text, or social media?” (daily, more than once a week, less than once a week, once or less a month) [31,50,51].

Participants access and use of technology was assessed by two survey items. The first assessed whether the respondent had a home computer (yes, no), while the second assessed if respondents had Internet access at home (yes, no) at home [31,50,51]. Participants also reported how often they checked email (daily, more than once a week, less than once a week, once or less a month) and if they had a cell phone where they could receive SMS/Text messages (yes, no). Respondents also reported if they used WhatsApp (yes, no) and social media (e.g., Facebook, Instagram, Pinterest) (yes, no). Lastly, respondents reported how often they check messages on WhatsApp, SMS/text, or social media” (daily, more than once a week, less than once a week, once or less a month) [31,50,51]. Finally, language preference was measured by the following question: “If you were to enroll in a health promotion program for parents of preschool-age children, what would be your preferred language(s) to receive such information?” (1 = Portuguese, 2 = Either Portuguese or English, 3 = English) [31].

### 2.5. Analysis

All analyses were performed using SAS 7.1 (SAS Institute). We calculated means and standard deviations for all continuous variables and frequencies and percent for categorical variables [31,50,51]. Responses for preference for informational content, intervention modality, and preferred language were dichotomized for analysis based on the distribution of the data [31,50,51]. We used Wilcoxon rank sum, Chi-square test, and Fisher’s exact test appropriately to examine if there were differences in mothers’ and fathers’ preference for informational content related to EBRBs (interested/very interested vs. not interested), intervention modality (completely agree vs. neutral/disagree), and language (Portuguese vs. Portuguese or English/English) [31,50,51].

## 3. Results

A total of 52 Brazilian immigrant parents participated in the study, 27 mothers and 25 fathers. All parents were born in Brazil and reported having lived in the U.S. for an average of 7.6 years (SD = 6.6 years) [31]. Approximately 93% (n = 48) were classified as having a low level of acculturation, and all reported Portuguese as their primary language. Moreover, while approximately 41% (n = 11) of mothers and 28% (n = 7) of fathers reported being overweight, the majority (92%, n = 48) perceived their child as having normal weight status [31]. Additional information on the study’s participants is presented in Table S1.

### 3.1. Parents’ Preferences for Informational Content

As shown in Table S2, most parents reported being interested/very interested in informational content focused on most of the assessed EBRBs. Regarding healthy eating behaviors, approximately 94% (n = 49) reported being interested/very interested in content related to reducing the consumption of unhealthy or junk foods (Table S2). Furthermore, 90.4% (n = 47) were interested/very interested in content related to increasing consumption of fruits and vegetables to 5 or more per day, 86.5% (n = 45) in content related to reducing SSB intake, and 69.2% (n = 36) in content related to promoting water consumption. Although more mothers than fathers reported being interested/very interested in content related to three of the four assessed healthy eating behaviors, these differences were significant only for reducing consumption of SSBs (88.9% vs. 56%; *p* = 0.008) and increasing intake of water (81.5% vs. 56%; *p* = 0.04).

Furthermore, about 91% (n = 47) of parents reported being interested/very interested in content related to increasing PA, 88.5% (n = 46) in content focused on limiting screen time, and 67.3% (n = 35) in content related to promoting healthy sleep. In contrast to healthy eating behaviors, as shown in Table S2, a greater proportion of fathers than mothers stated being interested/very interested in content related to increasing PA and limiting screen time to 2+ h per day. Nonetheless, these differences were not significant (Table S2).

### 3.2. Preferences for Intervention Modalities

Most parents (86.5%, n = 45) reported that they were interested/very interested in group sessions delivered by CHWs, with 96.2% (n = 25) of mothers and 80% (n = 20) of fathers endorsing this intervention modality (Table S3). Parents were also receptive to content being delivered via email (84.6%, n = 44), SMS/text (80.4%, n = 41), printed materials (69.2%, n = 36), group sessions led by peer parents (61.5%, n = 32), WhatsApp (51.9%, n = 27), and individual home visits by CHWs (50%, n = 26), with all these modalities being endorsed by half or more of the participating parents.

A greater proportion of mothers than fathers endorsed information content being delivered via group sessions led by CHWs (96.2% vs. 80%; *p* = 0.18), email (88.9% vs. 80%; *p* = 0.37), group sessions by peer parents (74.1% vs. 48%; *p* = 0.05), WhatsApp (59.3% vs. 44%; *p* = 0.27), and individual visits by CHWs (63% vs. 36%; *p* = 0.05). However, these differences were significant only for group sessions by peer parents and individual visits by CHWs (Table S3). In contrast, a greater proportion of fathers than mothers endorsed content being delivered via text/SMS (80% vs. 77.8%; *p* = 0.85) and printed materials (80% vs. 59.3%; *p* = 0.11). However, these differences were not significant.

Some intervention modalities were endorsed by less than half of the parents. These modalities included content delivered via Portuguese language websites (32.7%, n = 17), English language websites (23.1%, n = 12), social media (21.2%, n = 11), and telephone calls (19.2%, n = 10). Although a greater proportion of mothers than fathers endorsed information content delivered by these modalities, this difference was significant only for social media (33.3% vs. 8%, *p* = 0.03) (Table S3).

### 3.3. Communication Technology Access and Use

Nearly all parents (96.2%, n = 50) reported having access to a mobile phone where they could receive text messages, about 90% (90.4%, n = 47) reported using WhatsApp, and 85.2% (n = 39) reported checking WhatsApp messages daily. A significantly higher percentage of mothers than fathers (100% vs. 80%, *p* = 0.02) reported using WhatsApp and checking for text messages daily (85.2% vs. 56%, *p* = 0.003). In contrast, only 25% of respondents (n = 13) reported checking their email daily, with a higher proportion of mothers than fathers reporting they checked their email daily (37% vs. 12%, *p* = 0.04). Additionally, although 92.3% (n = 48) of parents reported having Internet access at home, only 63.5% (n = 33) reported having access to a computer at home. A higher proportion of mothers than fathers (77.8% vs. 48%, *p* = 0.03) reported having access to a home computer [31].

### 3.4. Language Preference

Finally, most parents (71.2%, n = 37) reported a preference for delivered information to be in Portuguese, while 23% (n = 12) indicated no preference, and 5.7% (n = 3) preferred information be delivered in English [31]. There were no significant differences in language preference between mothers (70.4%, n = 19) and fathers (72%, n = 18) (Table S4).

## 4. Discussion

The present study found important and significant differences between mothers’ and fathers’ preferences for intervention content and delivery modalities. As with previous studies with other Latino populations, a higher proportion of mothers than fathers in our study reported being interested/very interested in learning about most of the assessed EBRBs [26,54,55,56]. Interestingly, a higher percentage of fathers than mothers reported being interested/very interested in content related to children reducing consumption of junk food, promoting PA, and limiting screen time [31]. Although these differences were only significant for reducing the consumption of SSBs and increasing water consumption, they warrant further investigation. Parents were least interested in receiving information about promoting water consumption and adequate night sleep (>10 h/night). This finding suggests that there is a need for increased education regarding the importance of these two EBRBs, which are essential for preventing obesity and promoting children’s overall health and development [4,5,7,16].

In the current study, most parents preferred interventions involving direct interaction with CHWs (“promotores” or community health agents) with content delivered in Portuguese. CHWs are peer health educators who are trusted individuals from the community and share common characteristics with the priority population [57,58,59,60]. Previous research with Latino populations has successfully employed CHWs to deliver interventions to promote healthful EBRBs and prevent obesity [23,61,62]. Recent culturally tailored interventions delivered by CHWs designed to promote healthful EBRBs and prevent obesity in Latino children in the U.S., such as ANDALE Pittsburgh [23,24,62], Aventuras Para Niños [63], and La Vida Buena [56] have shown high acceptability and feasibility. Nonetheless, it should be noted that mothers accounted for most participants in these interventions, and these were conducted with Hispanic parents, not Brazilians [23,24,63]. In Brazil, CHWs (also known as “Agentes Comunitários de Saúde”) are an integral part of the government-sponsored child and family health programs that include home visits, and evidence suggests the effectiveness and acceptability of CHW-led child and adult health programs in Brazil [64,65]. This may partially explain why mothers and fathers participating in this study supported group sessions delivered by CHWs. In addition, most mothers favored group sessions delivered by peer parents. Combined with existing research findings, these findings suggest that interventions should consider offering group sessions and fostering social support by individuals of similar sociocultural backgrounds.

Email and SMS/text messages were the second and third most preferred delivery channels for receiving informational content. Although a higher proportion of parents favored email over SMS as a delivery channel, only a small percentage reported checking their email daily. In contrast, nearly all parents checked SMS/text messages daily. These findings suggest that SMS/text may be a feasible communication channel for intervention delivery for this population [50,51]. Previous studies indicate the acceptability and feasibility of using SMS/text as a practical and low-cost way to deliver health information to parents at home [66,67,68,69]. Therefore, future child health promotion and obesity prevention interventions should consider SMS/text messages as a potential channel for reaching Brazilian immigrant parents to communicate health messages to support healthful EBRBs [31]. Moreover, nearly all parents in the current study reported using WhatsApp, and more than 90% reported checking WhatsApp messages daily. WhatsApp is a popular cross-platform instant messaging application that facilitates communication, information sharing, and human interaction among Latin Americans, including Brazilians [36,69,70,71]. Notably, a greater proportion of mothers than fathers reported using and checking WhatsApp messages daily, which suggests that WhatsApp might be a good communication tool for prompting healthful EBRBs. Given that a high proportion of parents in this study reported using WhatsApp, it is likely a feasible and acceptable communication channel for the delivery of intervention content given that it is free of cost for exchanging text, image, video, and audio messages and should be considered in the development of future interventions for Brazilian immigrant families [69,70,71].

Our findings also revealed that most parents preferred intervention content be in their native language, Portuguese. This finding aligns with the acculturation levels of Brazilian immigrants participating in this study and previous studies [25,31,72] that document that Portuguese is the preferred and most used language by foreign-born Brazilians in the U.S. [31]. Previous studies with other minority immigrant populations suggest that language and communication can be barriers to parents understanding and engaging in interventions to promote healthful EBRBs, indicating the importance of interventions being linguistically tailored to meet the needs of specific ethnic minority immigrant populations, such as Brazilians [31,39,46,47,48,49,50,51,73,74].

Some study limitations include a small, convenience sample of Brazilian-born immigrant parents classified as having a low acculturation level living in a few cities in Massachusetts, which limits generalizability and should be considered when interpreting the study findings. In addition, participants may have had a heightened interest and awareness regarding the study topics. In addition, the present study did not include an assessment of the influence of some popular social media, such as TikTok, on parents perceptions of the importance of their children’s EBRBs [31]. Finally, as is often the case with formative research [32,33], the convenience sample in the present study is small, consequently limiting our ability to conduct additional analyses [31,50,51]. To address these limitations, future studies should include a larger sample size and Brazilian immigrant parents from other communities in the U.S. [31]. Nonetheless, the present study provides new information about the preference of Brazilian immigrant parents related to healthy eating and 24 h movement behaviors that may be used to design interventions to promote healthful EBRBs and prevent childhood obesity in preschool-age children in Brazilian immigrant families in the U.S. [31].

## 5. Conclusions

This formative research is the first to examine Brazilian immigrant parents’ preferences for informational content, delivery modality, and access and use of communication technology to develop a family-based intervention to promote healthful EBRBs and prevent child obesity [31]. Findings provide important information that can help guide the development of future family-based interventions tailored to meet the specific needs of this growing immigrant population in the U.S. [31].

Future steps by the research team include using findings to inform the design of a linguistic and cultural-sensitive family-based intervention to promote healthful EBRBs of preschool-age children in Brazilian immigrant families [31]. The intervention will integrate different communication strategies and be pilot tested to assess its acceptability and effectiveness. Intervention modalities, including direct contact, interactive interventions delivered by CHWs combined with mHealth, including SMS/text messages and WhatsApp messages, and printed materials, will be considered as methods to enable the sharing of information to promote Brazilian immigrant parents’ increased knowledge, skills, and practices needed to create a home environment supportive of their preschool-age children’s healthful EBRBs [50,51,66,67,68,69]. As behavior change and promoting healthful behaviors are complex, future interventions will likely need multiple components as one single delivery channel may not be sufficient to effectively promote EBRBs among children of Brazilian immigrant families in the U.S. [31,50,51].

## Supplementary Materials

The following supporting information can be downloaded at: https://www.mdpi.com/article/10.3390/ijerph20064817/s1; Table S1: Sociodemographic and acculturation characteristics of study participants; Table S2: Participants’ preferences for informational content for intervention modalities designed to promote healthful energy balance-related behaviors of their preschool-aged children; Table S3: Participants’ preferences for intervention modality for development of family-based intervention to promote healthful energy balance-related behaviors of their preschool-aged children; Table S4: Participants’ language preferences for receipt of information and communication technology access and frequency of use.

## Abbreviations

CHWs: community health workers; EBRBs: energy balance-related behaviors; PA: physical activity; SASH: Short Acculturation Scale for Hispanics; SSBs: sugar-sweetened beverages; U.S.: United States
