# Supplementary material for: Brazilian Immigrant Parents’ Preferences for Content and Intervention Modalities for the Design of a Family-Based Intervention to Promote Their Preschool-Age Children’s Healthful Energy Balance-Related Behaviors
Source: Int J Environ Res Public Health. 2023 Mar 9;20(6):4817. doi: 10.3390/ijerph20064817 (PMC10048827; doi:10.3390/ijerph20064817)
Supplement: Supplementary file 1 [file ijerph-20-04817-s001.zip › ijerph-2160715-supplementary.docx]

**Table S1.** Sociodemographic and acculturation characteristics of study participants (N=52).

| **Parents’ Characteristics** | | **Mothers**  **N = 27 (%)** | **Fathers**  **N = 25 (%)** | **Total Sample**  **N = 52 (%)** | ***p-*value** |
| --- | --- | --- | --- | --- | --- |
| **Sociodemographic variables** | | | | | |
| **Age (mean SD)^1^** | | 34.7 (5.6) | 36.1 (5.7) | 35.4 (5.6) | 0.36 |
| **Marital status** | |  |  |  |  |
| Married/Cohabitating | | 26 (96.3) | 25 (100.0) | 51 | 1.00 |
| Divorced | | 1 (3.7) | 0 | 1 |  |
| **Educational attainment** | |  |  |  |  |
| < High school diploma | | 3 (11.1) | 6 (24.0) | 9 (17.3) | 0.40 |
| High school diploma | | 9 (33.3) | 9 (36.0) | 18 (34.6) |  |
| > High school | | 15 (55.6) | 10 (40.0) | 25 (48.1) |  |
| **Household income** | |  |  |  |  |
| < US40,000/year | | 10 (37.0) | 13 (52.0) | 23 (44.2) | 0.30 |
| > US40,000/year | | 8 (29.6) | 5 (20.0) | 13 (25.0) |  |
| Declined to report/Don’t Know^2^ | | 9 (33.4) | 7 (28.0)^1^ | 16 (30.8) |  |
| **Acculturation variables** | | | | | |
| **Years living in the United States** | | 7.3 (6.2) | 7.9 (7.0) | 7.6 (6.6) | 0.80 |
| **SASH Score** | |  |  |  |  |
| < 2.99 | | 26 (96.3) | 22 (88.0) | 48 (92.2) | 0.34 |
| > 2.99 | | 1 (3.7) | 3 (12.0) | 4 (7.8) |  |
| **Weight status variables** |  | | | | |
| **Weight status**  Normal weight | | 14 (51.9) | 18 (72.0) | 32 (61.5) | 0.19 |
| Overweight | | 11 (40.7) | 7 (28.0) | 18 (34.6) |  |
| Obese | | 2 (7.4) | 0 | 2 (3.9) |  |
| **Has your doctor expressed concern about your weight status?** | |  |  |  |  |
| Yes | | 6 (22.2) | 1 (4) | 7 (13.5) | 0.10 |
| No | | 20 (74.1) | 22 (88) | 42 (80.7) |  |
| Missing | | 1 (3.7) | 2 (8) | 3 (5.8) |  |
| **Child characteristics** | | | | | |
| **Age** (mean; SD^2^) | | 3.1(1.2) | 3.2 (1.8) | 2.9 (1.6) | 0.80 |
| **Sex** | |  |  |  |  |
| Male | | 13 (48.1) | 11 (44.0) | 24 (46.2) | 0.93 |
| Female | | 14 (51.9) | 14 (56.0) | 28 (53.8) |  |
| **Place of nativity** | |  |  |  |  |
| United States | | 15 (55.6) | 16 (64.0) | 31 (59.6) | 1.00 |
| Brazil | | 12 (44.4) | 9 (36.0) | 21 (30.4) |  |
| **Weight status (as reported by parent)** | |  |  |  |  |
| Underweight | | 2 (7.4) | 0 | 2 (3.9) | 0.92 |
| Normal weight | | 23 (85.2) | 25 (100.0) | 48 (92.2) |  |
| Overweight | | 2 (7.4) | 0 | 2 (3.9) |  |

^1^Standard deviation; ^2^Only fathers declined to report.

**Table S2.** Participants’ preferences for informational content for intervention modalities designed to promote healthful energy

balance-related behaviors of their preschool-aged children.

| **Energy Balance-Related Behaviors** | **Mothers**  **N = 27 (%)** | **Fathers**  **N = 25 (%)** | **Total Sample**  **N = 52 (%)** | ***p*-value** |
| --- | --- | --- | --- | --- |
| **Promotion of Healthful Eating Behaviors** | | | | |
| **Reduce consumption of unhealthful/”junk” foods** | | | | |
| Interested/Very Interested | 25 (92.6) | 24 (96.0) | 49 (94.2) | 0.60 |
| Not interested | 2 (7.4) | 1 (4.0) | 3 (5.8) |  |
| **Increase consumption of fruits and vegetables** | | | | |
| Interested/Very Interested | 26 (96.3) | 21 (84.0) | 47 (90.4) | 0.13 |
| Not interested | 1 (3.7) | 4 (16.0) | 5 (9.6) |  |
| **Reduce consumption of sugar-sweetened beverages** | | | | |
| Interested/Very Interested | 24 (88.9) | 14 (56.0) | 45 (86.5) | 0.008** |
| Not interested | 3 (11.1) | 11 (44.0) | 7 (13.5) |  |
| **Increase consumption of water** | | | | |
| Interested/Very Interested | 22 (81.5) | 14 (56.0) | 36 (69.2) | 0.04* |
| Not interested | 5 (18.5) | 11(44.0) | 16 (30.8) |  |
| **Promotion of Healthful 24-Movement Behaviors** | | | | |
| **Increase physical activity levels (> 60 minutes/day)** | | | | |
| Interested/Very Interested | 24 (88.9) | 23 (92.0) | 47 (90.4) | 0.71 |
| Not interested | 3 (11.1) | 2 (8.0) | 5 (9.6) |  |
| **Limit screen time (< 2 hours/day)** | | | | |
| Interested/Very Interested | 23 (85.2) | 23 (92.0) | 46 (88.5) | 0.44 |
| Not interested | 4 (14.8) | 2 (8.0) | 6 (11.5) |  |
| **Promote adequate healthy sleep (> 10 hours/night)** | | | | |
| Interested/Very Interested | 20 (74.0) | 15 (60.0) | 35 (67.3) | 0.28 |
| Not interested | 7 (26.0) | 10 (40.0) | 17 (32.7) |  |

*p*-value was calculated between “very interested/interested” and “not interested” groups p < 0.05*; p < 0.01**.

**Table S3.** Participants’ preferences for intervention modality for development of family-based intervention to promote

healthful energy balance-related behaviors of their preschool-aged children.

| **Intervention Modality for Delivery of Information** | **Mothers**  **N = 27 (%)** | **Fathers**  **N = 25 (%)** | **Total Sample**  **N = 52 (%)** | ***p*-value** |
| --- | --- | --- | --- | --- |
| **Group sessions delivered by CHW** | | | | |
| Completely agree | 25 (92.6) | 20 (80.0) | 45 (86.5) | 0.18 |
| Neutral/Disagree | 2 (7.4) | 5 (20.0) | 7 (13.5) |  |
| **E-mail** | | | | |
| Completely agree | 24 (88.9) | 20 (80.0) | 44 (84.6) | 0.37 |
| Neutral/Disagree | 3 (11.1) | 5 (20.0) | 8 (15.4) |  |
| **Text or SMS** | | | | |
| Completely agree | 21 (77.8) | 20 (80.0) | 41 (78.8) | 0.85 |
| Neutral/Disagree | 6 (22.2) | 5 (20.0) | 11 (21.2) |  |
| **Printed materials (e.g., pamphlets, booklets, etc.)** | | | | |
| Completely agree | 16 (59.3) | 20 (80.0) | 36 (69.2) | 0.11 |
| Neutral/Disagree | 11 (40.7) | 5 (20.0) | 16 (30.3) |  |
| **Group sessions delivered by peer-parents** | | | | |
| Completely agree | 20 (74.1) | 12 (48.0) | 32 (61.5) | 0.05* |
| Neutral/Disagree | 7 (25.9) | 13 (52.0) | 20 (38.5) |  |
| **WhatsApp** | | | | |
| Completely agree | 16 (59.3) | 11 (44.0) | 27 (51.9) | 0.27 |
| Neutral/Disagree | 11 (40.7) | 14 (56.0) | 25 (38.1) |  |
| **Individual home visits by CHW** | | | | |
| Completely agree | 17 (63.0) | 9 (36.0) | 26 (50.0) | 0.05* |
| Neutral/Disagree | 10 (37.0) | 16 (64.0) | 26 (50.0) |  |
| **Portuguese language website** | | | | |
| Completely agree | 12 (44.4) | 5 (20.0) | 17 (32.7) | 0.06 |
| Neutral/Disagree | 15 (55.6) | 20 (80.0) | 35 (67.3) |  |
| **English language website** | | | | |
| Completely agree | 8 (29.6) | 4 (16.0) | 12 (23.1) | 0.24 |
| Neutral/Disagree | 19 (70.4) | 21 (84.0) | 40 (76.9) |  |
| **Social media (e.g., Facebook, Instagram, Pinterest)** | | | | |
| Completely agree | 9 (33.3) | 2 (8.0) | 11 (21.2) | 0.03* |
| Neutral/Disagree | 18 (66.7) | 23 (92.0) | 41 (78.8) |  |
| **Telephone calls by community health worker (CHW)** | | | | |
| Completely agree | 7 (25.9) | 3 (12.0) | 10 (19.2) | 0.21 |
| Neutral/Disagree | 20 (74.1) | 22 (88.0) | 42 (80.8) |  |

*p*-value was calculated between “completely agree” and “neutral + disagree” groups; **p* < 0.05

**Table S4.** Participants’ language preferences for receipt of information and communication technology access and frequency of use.

|  | **Mothers**  **N = 27 (%)** | **Fathers**  **N = 25 (%)** | **Total Sample**  **N = 52 (%)** | ***p*-value** |
| --- | --- | --- | --- | --- |
| **In what language would you prefer to receive information?^1^** | | | | |
| Portuguese | 19 (70.4) | 18 (72.0) | 37 (71.2) | 0.90 |
| Either Portuguese or English | 7 (25.9) | 5 (20.0) | 12 (23.1) |  |
| English | 1 (3.7) | 2 (8.0) | 3 (5.7) |  |
| **Do you have access to a computer at home?** | | | | |
| Yes | 21 (77.8) | 12 (48.0) | 33 (63.5) | 0.03* |
| No | 6 (22.2) | 13 (52.0) | 19 (36.5) |  |
| **Do you have access to Internet at home?** | | | | |
| Yes | 25 (92.6) | 23 (92.0) | 48 (92.3) | 0.93 |
| No | 2 (3.8) | 2 (8.0) | 4 (7.7) |  |
| **How often do you check your e-mail^2^** | | | | |
| Daily | 10 (37.0) | 3 (12.0) | 13 (25.0) | 0.04* |
| More than once a week | 12 (44.4) | 10 (40.0) | 22 (43.3) |  |
| Less than once a week | 3 (11.1) | 5 (20.0) | 8 (15.4) |  |
| Once or less a month | 2 (7.4) | 7 (28.0) | 9 (17.3) |  |
| **Do you have a mobile telephone where you can receive SMS/Text?** | | | | |
| Yes | 27 (100.0) | 23 (92.0) | 50 (96.2) | 0.23 |
| No | 0 | 2 (8.0) | 2 (3.8) |  |
| **Do you use WhatsApp?** | | | | |
| Yes | 27 (100) | 20 (80.0) | 47 (90.4) | 0.02* |
| No | 0 | 5 (20.0) | 5 (9.6) |  |
| **Do you use social media (e.g., Facebook, Instagram, Pinterest)** | | | | |
| Yes | 27 (100) | 15 (80.0) | 42 (80.8) | 0.0003** |
| No | 0 | 10 (20.0) | 10 (19.2) |  |
| **How often do you check messages on your WhatsApp, SMS/text, or social media^3^** | | | | |
| Daily | 25 (85.2) | 14 (56.0) | 33 (85.0) | 0.003** |
| More than once a week | 2 (7.4) | 9 (36.0) | 11 (21.2) |  |
| Less than once a week | 0 | 2 (8.0) | 2 (3.8) |  |

^1^*p*-value was calculated between “Portuguese” vs. “Either Portuguese or English + English

^2^*p*-value was calculated between “daily” vs. “all other options”

^3^ *p*-value was calculated between “daily” vs. “all other options”; p < 0.05*; p < 0.01**.
